# Simulation-a new educational paradigm?

**DOI:** 10.7555/JBR.27.20120107

**Published:** 2013-02-10

**Authors:** Mojca Konia, Aubrey Yao

**Affiliations:** a Department of Anesthesiology, University of Minnesota, Twin Cities, MN 55455, USA;; b Department of Anesthesiology and Pain Medicine, University of California Davis, Sacramento, CA 95817, USA.

**Keywords:** simulation, education, outcomes

## Abstract

Simulation is a modern educational tool that has recently gained in the field of medical education. The use of simulation continues to expand, and studies evaluating the effectiveness of simulation-based medical education are ongoing. The history of medical education and adult educational theory are reviewed, and the details of effective simulation techniques are described. Finally, outcomes of simulation-based medical education are summarized.

## INTRODUCTION

Over the past century, medical education in North America has been delivered based on the principles described and implemented by Abraham Flexner in 1910. He recommended that medical school education consist of two years of basic science education and two years dedicated to clinical experience[1]. This was accomplished primarily in the form of didactic sessions and a clinical mentorship resembling an apprenticeship. Medical education has further evolved by the successful integration of problem-based learning. The effectiveness of medical education has not been thoroughly investigated for its educational outcomes, nor have there been high-quality studies comparing the effectiveness of educational methods. A report by the Institute of Medicine entitled “To Err is Human” highlighted deficiencies in modern healthcare systems and encouraged federal agencies to reevaluate the medical education system[2]. An improved evidence-based model of medical education emerged in response to this report, and the door has been further opened for the introduction and development of new educational tools.

Like the aviation industry, medicine is perceived as a technologically innovative and high-stakes profession. Technological advances require healthcare providers to develop new psychomotor and visual-spatial skill sets[3]. However, progressive limitations on resident work hours have made it increasingly difficult to provide an adequately diverse and comprehensive clinical education. Incorporation of simulation into medical training holds great promise in improving the overall quality and safety of patient care in the face of these challenges.

## EDUCATION WITH SIMULATION

Dr. Gaba, a pioneer of simulation in medical education, defined simulation as an instructional process that substitutes real patient encounters with artificial models, live actors, or virtual reality patients[4]. In their review of medical simulation, Carroll and Messenger observed that simulation has evolved from an esoteric tool with limited utility (used solely to acquire psychomotor skills) to a powerful modality that is used comprehensively to promote learning, facilitate training, and evaluate performance[3]. As an assessment tool, simulation can range from basic levels of performance to high-stakes board certification. The same authors also highlighted numerous advantages of simulation education: attaining basic levels of competence before patient encounters, learning to avoid errors, treating adverse events, and practicing and performing tasks early in training. Furthermore, simulation provides a solution to the conflict between education and service, corrects for case-mix inequality, and allows for comparison across training programs and even countries[3].

Learners in medical education at all levels are typically adult learners. Adult learners characteristically want to know why they are learning, prefer to problem-solve, have diverse backgrounds, possess knowledge assumptions based on prior experiences, and demand an educational process that is active and immediately applicable[5]. Simulation fulfills these criteria and offers an individualized learner experience in which the progression from novice, advanced beginner, competent, proficient, to master level can (and should) be observed[6]. However, progress through these stages only occurs if certain conditions are fulfilled: feedback, deliberate practice, curriculum integration, outcome measurement, simulation fidelity, skill acquisition and maintenance, mastery learning, transfer to practice, team training, high-stakes testing, instructor training, and educational and professional context[7]. Of these, feedback, deliberate practice and curriculum integration are of particular importance.

Debriefing is the process in which instructors and learners reexamine the clinical scenario through reflective discussion. Critical Incident Stress Debriefing (CISD) was developed by Mitchell with the intent of helping health care workers better manage the stress and emotional tension of critical medical events by reviewing facts, events and reactions with a facilitator [8]. The facilitator plays an essential role in guiding the participants to a more systematic reflection of the event. As Fanning and Gaba explained in their review, the learner (who is in the so-called hot seat) may find it very difficult to transition from the experience of the event to a thoughtful analysis and plan for potential improvement and modification of behavior[9]. An effective facilitator will help participants by developing a supportive climate in an environment of trust in which learning occurs without the stressful and counterproductive fear of peer judgment[10].

Debriefing consists of three phases: recollection of scenario events and participant actions, exploration of the feelings and emotions experienced, and analysis and application to real-life situations[11]. Debriefing is the most vital component of the simulation experience -in fact, it has been said that simulation is undertaken in order to debrief. Savoldelli and coauthors found that simulation without debriefing led to no improvement in nontechnical skills compared to simulation with verbal/oral feedback[12]. Edelson et al. studied the impact of feedback on in-hospital CPR performance using a novel debriefing protocol (resuscitation with actual performance integrated debriefing-RAPID) enhanced by objective data from a CPR-sensing and feedback-enabled defibrillator. The CPR performance of simulator-trained residents was compared with the performance of a historical resident cohort. The simulator-trained group displayed significantly better CPR performance than the historical cohort on a variety of clinically meaningful measures including return of spontaneous circulation. From these illustrations, we see that simulation-based medical education (SBME) with potent feedback has a clear impact on trainee clinical behavior and even patient outcomes[13].

## APPLICATION OF SIMULATORS

**Fig. 1:**
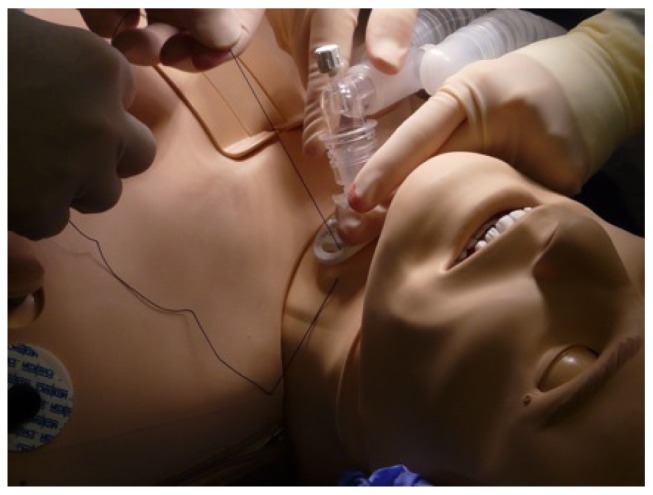
Insertion of tracheostomy can be practiced by learners in a safe simulation environment without exposing actual patients to any risk

**Fig. 2:**
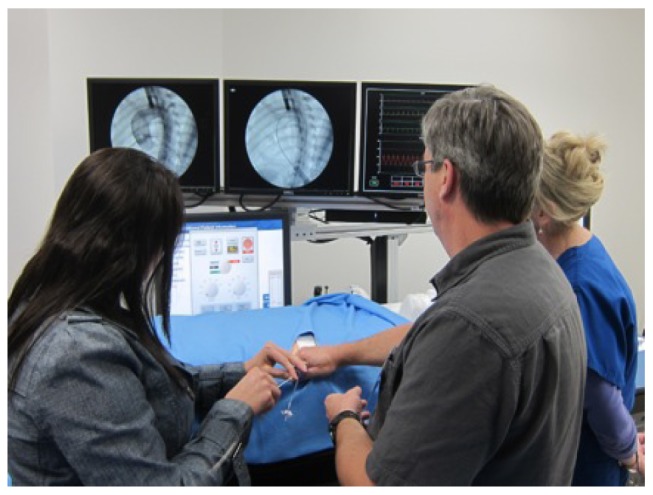
New technologies and skills can be practiced in a simulated environment prior to performing the procedure on the patient, as, for example, transcatheter aortic valve implantation.

The simulation experience stimulates critical thinking, decision-making and clinical judgment. It provides the adult learner with active, experiential learning and enables participants to practice rare or emergent medical conditions in a safe, non-threatening environment. Learners can make mistakes without fear of injuring an actual patient (***Fig. 1***). It allows for development of specific skills prior to interaction with the patient and has been shown to decrease the incidence of complications, for example, with central line placement[15]. It can improve communication skills, which are recognized as a core competency by organizations such as the Accreditation Council for Graduate Medical Education (ACGME), which is responsible for the accreditation of postgraduate medical training programs in the United States. Simulation can also be used to analyze teamwork and the other human factors that play key roles in the management of critical situations, and it is a mainstay in crisis resource management training. Furthermore, simulation can provide a medium for testing new procedures and devices (***Fig. 2***). Simulation is becoming an integral part of medical education, and it is likely that its use will continue to expand as technology advances.

**Fig. 3:**
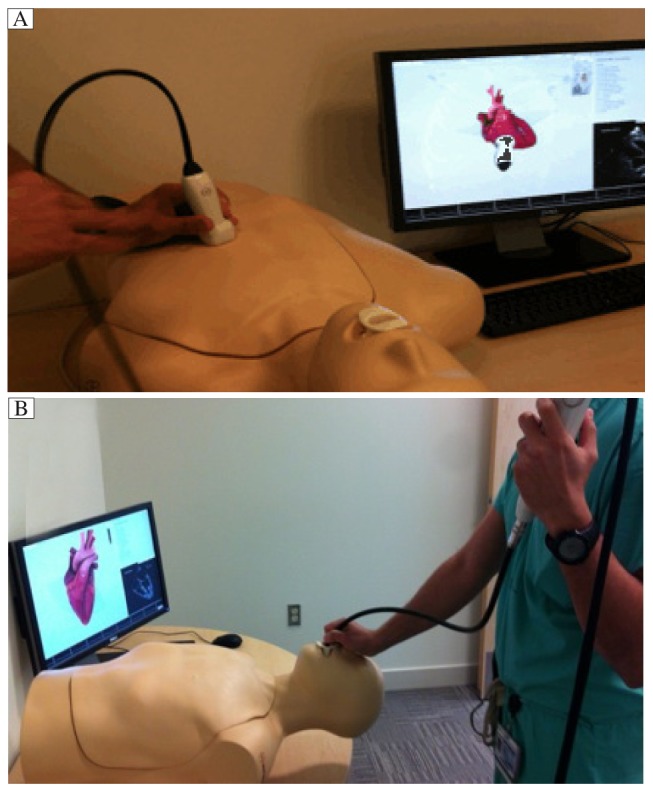
Interactive video-assisted simulators can be used to learn basic skills of transthoracic (A) and transesophageal (B) echocardiogram.

Simulators in current use include: an actor assuming a specific role (currently utilized for objective structured clinical examinations); animal models; human cadavers; interactive video-based simulation (e.g. bronchoscopy, echocardiography) (***Fig. 3***); task trainers used to practice a particular procedure (e.g. endoscopy); and full-environment simulation using high-fidelity mannequin simulators[16].

When utilizing simulation as an educational tool, the following must be considered:

- Careful analysis of the learning domain of the Bloom's taxonomy being addressed: cognitive, psychomotor, affective, or a combination of the three[17].
- Understanding of the participant's level in a specific learning domain (Bloom's taxonomy) as well as the desired level to be achieved. In the cognitive domain, knowledge and comprehension do not require simulation. On the other hand, application and analysis can be easily taught, observed and debriefed after a simulated experience. The use of simulation for the planning of a new procedure is an example of simulation being used at the level of synthesis and perhaps even evaluation. Simulation has been used for the development of the affective domain of learning. Training of team, leadership, and organizing behaviors can be successfully done with the use of simulation. The utility of simulation for teaching different visual-spacial skills and for practicing procedures are well-documented in the literature. However, teaching with simulation requires a careful evaluation of whether or not the learner has achieved complex overt response -a level of psychomotor skills that they will transfer from the simulation laboratory into clinical practice.
- The types of simulators available and the most effective type for teaching the particular objectives[18].

## DOES IT WORK?

Simulation has been proposed as an educational modality that improves safety in High Reliability Organizations and has been extensively utilized in the aviation industry. In medicine, simulation has been used for routine training for emergencies, developing teamwork, establishing an environment for discussing errors without punishment, evaluating and testing new procedures for safety, assessing competence, testing usability of devices, investigating human performance, and providing skills training for novices outside of the clinical environment[19]. Participants should be satisfied with the learning experience and with the learning that should have occurred, but the primary objective is to achieve behavior change whereby knowledge, skills and attitudes have transferred from the simulation environment into clinical practice and have resulted in improved patient outcomes[20]. Ultimately, the goal of simulation education is to increase patient safety and to improve outcomes.

**Fig. 4:**
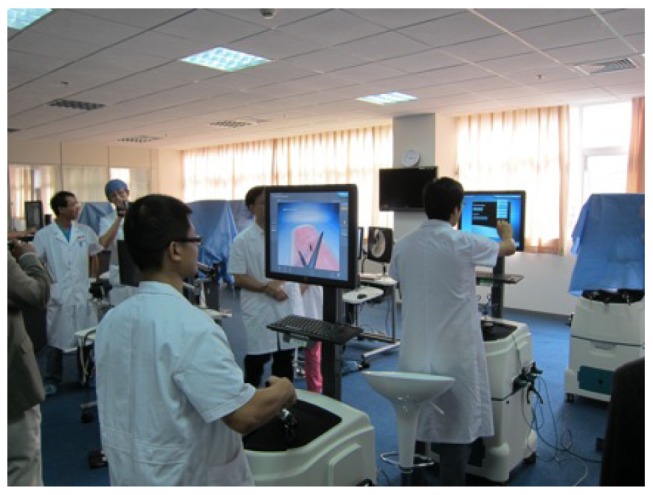
Task trainers for skills such as endoscopy are in extensive use already. They allow learners to practice specific visual-spatial skills required for endoscopic surgery.

When assessing the value of an educational experience, one needs to consider Miller's four levels of clinical competence assessment n. An individual moves from knowledge through integrated knowledge, competence and finally to performance; Miller called these four stages 'Knows, Knows How, Shows How, and Does[21]. 'Nishiaki proposed a framework that consisted of four key levels of assessing the effectiveness of simulation-based education[19]:

- Self-efficacy, which reflects one's beliefs about the ability to perform actions (Knows and Knows How). Crisis resource management courses have demonstrated effectiveness on self-efficacy for emergency medicine residents[22] and critical-care fellows[23]. However, caution must be advised because increases in self-efficacy do not necessarily translate into increased competency[24]. The actual ability to perform a particular task is poorly correlated with the learner's self-assessment, and learners at lower stages of the Dreyfus model are particularly inaccurate at assessing their actual ability to perform a specific task[25];
- Competence, which demonstrates one's ability to perform a task in the simulated environment (Miller's Shows). Procedural simulators and high-fidelity simulations have been shown to increase trainee competence in emergency airway management[26]. There is also good evidence that clinical competence can be measured with reliability and validity as demonstrated by studies by Murray et al. and by Boulet et al.[27], [28];
- Operational performance, which measures how competent care in a simulated environment translates into clinical care (Miller's Does). Numerous studies investigating the effect of simulation training on laparoscopic skills of residents demonstrate the transfer of skills from the simulation laboratory into the clinical environment[29],[30] (***Fig. 4***).
- Patient outcome, which analyzes the positive effects of learning for patient care. The impact of simulation education on actual patient safety is of paramount importance. In recent years, evidence has accumulated that supports the beneficial effects of SBME[30].

Two questions related to patient outcomes have been explored in the literature. The first evaluates whether education with simulation improves knowledge, skills and attitudes and thus translates into a better patient outcome. The second question explores the persistence of the educational effects.

Several systematic reviews and meta-analyses have examined these issues. Cook and coauthors evaluated the outcomes of technology-enhanced simulation training for health professions in comparison to no intervention[31]. The authors identified 609 studies that included 35,226 learners. They demonstrated a pooled effect size of 1.20 (95% CI, 1.04-1.35) for medical knowledge, 1.14 (95% CI, 1.03-1.25) for time skills, 1.09 (95% CI, 1.03-1.16) for process skills, 1.18 (95% CI, 0.98-1.37) for product skills, 0.79 (95% CI, 0.47-1.10) for time behaviors, 0.81 (95% CI, 0.66-0.96) for other behaviors, and 0.50 (95% CI, 0.34-0.66) for effects on patient outcomes. In other words, simulation-based education significantly impacts knowledge, skills and behaviors and has a moderate effect on patient outcomes.

Ross and coauthors reviewed studies of simulation-based education in anesthesia-related journals[32]. They identified 320 papers, out of which 11 examined patient outcomes and only two were controlled randomized trials which used blinded assessors[33],[34]. Those two studies demonstrated that simulation training led to higher clinical and non-technical skills levels than didactic teaching.

Another quantitative meta-analysis by McGaghie and coauthors reviewed simulation-related publications over two decades (1990-2010)[35]. The four inclusion criteria used to select the pool of eligible studies for the final analysis were as follows: each study had to (1) feature SBME with deliberate practice as an educational intervention; (2) have an appropriate comparison group featuring traditional, clinical education or a pre-intervention baseline measurement for single group designs; (3) assess trainee skill acquisition rather than knowledge or attitudes; (4) present sufficient data to enable effect size (ES) calculation. Fourteen studies were identified that met inclusion criteria and the comparative effectiveness of SBME compared to traditional medical education was 0.71 (95% CI, 0.65-0.76; *P* < 0.001).

Besides the effect SBME can have on patient management skills, an important aspect of SBME resides in interdisciplinary team training. By analyzing existing teamwork patterns and communication principles, inefficient practices can be uncovered and corrected. Frequently, the cause of adverse events can be related to institutional policies and their implementation. Simulation team training can be utilized to identify system-level, specialty-specific practices, policies or procedures that could adversely affect patient care[36].

Skills acquisition longevity is another important aspect of any educational effort. Do the learners maintain their skills levels or do they deteriorate rapidly following the educational experience? Several recent studies have shown positive effects in terms of long-term effects. Wayne and coauthors have shown that ACLS skills acquired by internal medicine residents in the simulation laboratory persist for up to 14 months post-training[37]. Similarly, Crofts et al. demonstrated that acquired skill at managing shoulder dystocia in obstetrics is largely maintained up to 12 months post-SBME training among midwives and doctors in the UK[38]. Boet and coauthors have shown effects to last for 1 year[39].

## CONCLUSION

Simulation-based education has been in use for several decades and its usage is likely to continue expanding. Evolving public expectations about the safety of medicine (and a general unwillingness to be used for practice) as well as decreasing resident patient contact hours are some of the factors involved in an increased need for SBME. Simulation training is a proven training modality in other high-risk industries, and it should be increasingly utilized in the high-stakes field of medicine. Studies support its positive effects on learner knowledge, skills and behaviors, and patient outcomes by translating skills from the simulated environment to the clinical environment.
